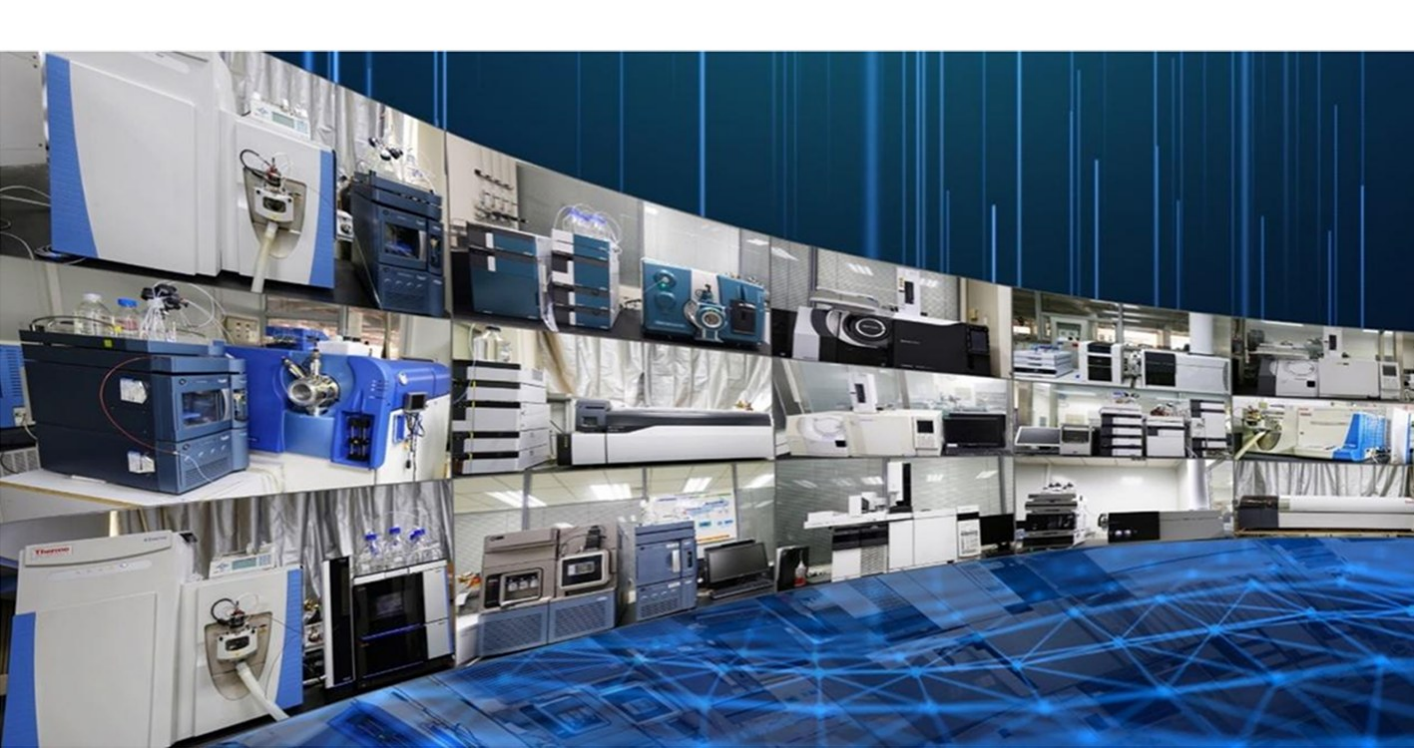# 基于色谱-质谱联用的暴露组学分析方法和研究范式的新进展

**DOI:** 10.3724/SP.J.1123.2023.12001

**Published:** 2024-02-08

**Authors:** Lei YOU, Guohao SUN, Di YU, Xinyu LIU, Guowang XU

**Affiliations:** 1.中国科学院大连化学物理研究所, 中国科学院分离分析化学重点实验室, 辽宁省代谢组学重点实验室, 辽宁 大连 116023; 1. Liaoning Province Key Laboratory of Metabolomics, CAS Key Laboratory of Separation Science for Analytical Chemistry, Dalian Institute of Chemical Physics, Chinese Academy of Sciences, Dalian 116023, China; 2.中国科学院大学, 北京 100049; 2. University of Chinese Academy of Sciences, Beijing 100049, China

**Keywords:** 色谱-质谱, 暴露组学, 代谢组学, 多组学关联研究, 综述, chromatography-mass spectrometry, exposomics, metabolomics, multi-omics association study, review

## Abstract

人类疾病的发生、发展受环境因素和遗传因素的共同影响,仅从遗传基因角度开展研究,不能有效地揭示疾病的发生机制,因而近年来环境因素对疾病发生、发展的影响受到了学界的广泛关注。暴露组学关注个体一生中所有暴露因素的测量,以及这些暴露因素与疾病建立联系的机制,为推动人类健康与环境因素之间的关系研究提供了新思路。由于外源性化学物质具有数量众多、理化性质各异、在体内含量极低等特性,对其进行测量会面临诸多挑战。基于色谱-质谱联用的暴露组分析技术兼具色谱的高效分离能力和质谱的高分辨率、高灵敏度特性,可以实现暴露组的高覆盖、高通量、高灵敏度检测,已成为暴露组学研究的主要分析技术。基于色谱-质谱联用的暴露组学分析方法主要包括靶向定量分析、可疑物筛查和非靶向筛查。除此之外,为了探究环境暴露与疾病发生、发展的关系,研究者发展了包括暴露组关联研究、混合暴露研究以及暴露组学与多组学(基因组学、转录组学、蛋白质组学、代谢组学)关联研究等的多种研究范式,这些方法的出现为暴露组学研究带来了空前的发展前景。本文综述了基于色谱-质谱联用技术的暴露组学分析方法和暴露组学研究范式,并对暴露组学的未来发展进行了展望。

## Abstract (en)

The occurrence and development of human diseases are influenced by both genetic and environmental factors. Research models that describe disease occurrence only from the perspective of genetics present certain limitations. In recent years, effects of environment factors on the occurrence and development of diseases have attracted extensive attentions. Exposomics focuses on the measurement of all exposure factors in an individual’s life and how these factors are related to disease development. Exposomics provides new ideas to promote studies on the relationship between human health and environmental factors. Environmental exposures are characterized with different physical and chemical properties, as well as very low concentrations in vivo, which contribute great challenges in the comprehensive measurement of chemical residues in the human body. Chromatography-mass spectrometry-based technologies combine the high-efficiency separation ability of chromatography with the high resolution and sensitive detection characteristics of mass spectrometry; the combination of these techniques can achieve the high-coverage, high-throughput, and sensitive detection of environmental exposures, thus providing a powerful tool for measuring chemical exposures. Exposomics-analysis methods based on chromatography-mass spectrometry mainly include targeted quantitative analysis, suspect screening, and non-targeted screening. To explore the relationship between environmental exposure and the occurrence and development of diseases, researchers have developed research paradigms, including exposome wide association study, mixed-exposure study, exposomics and multi-omics (genome, transcriptome, proteome, metabolome)-association study, and so on. The emergence of these methods has brought about unprecedented developments in exposomics studies. In this manuscript, analytical methods based on chromatography-mass spectrometry, exposomics research paradigms, and their relevant prospects are reviewed.

人类的健康或疾病状态受环境因素和遗传因素的共同影响。全基因组关联研究(genome wide association study, GWAS)表明,仅有10%~20%的疾病能够由基因变异来解释^[1,2]^。瑞典家庭癌症数据库数据显示,在15种常见的癌症中,仅有约10%的致癌风险来自遗传因素,而更多的风险来自于环境暴露以及遗传与环境暴露间的相互作用^[1,3,4]^。为了能够更加深入地研究环境暴露与人类健康或疾病状态之间的关系,研究者们提出了暴露组的概念。

暴露组是指一个人从出生至生命结束全过程中各种暴露的总和,其能从真正意义上探讨污染暴露与人体健康和疾病之间的关系,并揭示这种关联背后的内在本质。暴露组学关注个体一生中所有暴露因素的测量,以及这些暴露因素与疾病建立联系的机制^[5]^。暴露组概念的提出促进了以组学为手段的暴露与疾病研究的发展,这类研究采用高通量的组学技术来分析血液和尿液等生物基质中内、外源性有害物质的含量差异及变化趋势,从而揭示这些物质与疾病发生、发展之间的关系。

暴露组关联研究(exposome wide association study, EWAS)是探究环境暴露与疾病发生、发展关系过程中所采用的一种重要的研究范式,其能够对未知条件下的暴露情况进行评估。在进行EWAS时,需要确定暴露变量和结局,再根据多种暴露变量与疾病的相关性筛选出重要的暴露变量,从而实现关键暴露因子的识别^[6]^。在EWAS方法的基础上,Rappaport等^[7]^利用两阶段方法来研究疾病相关的重要暴露因素:第一阶段,比较疾病组与对照组血液/尿液中暴露组的差异,发现并鉴定特征性的化学物质,确定其与疾病的关联;第二阶段,在大规模血液/尿液样本中验证这些化学物质用作暴露标志物或疾病恶化标志物的可靠性。该方法能够从众多的内、外源性物质中发现重要的分析对象,有利于锁定真正的风险因子和有应用前景的预警标志物。

本文对基于色谱-质谱联用技术的暴露组学分析方法及研究范式的进展进行了介绍。首先综述了基于色谱-质谱的暴露组学分析方法进展,随后围绕环境因素导致的不良健康效应问题,概括了以暴露组为核心的暴露组关联研究、混合暴露研究及暴露组学与多组学(基因组学、转录组学、蛋白质组学、代谢组学)关联研究等研究范式,最后对暴露组学分析方法及研究范式的未来发展进行了展望。

## 1 基于色谱-质谱联用的暴露组学分析方法

暴露物是指个体在其生命过程中所接触到的各种物质,这些物质可能来自于环境、食品、空气、水等。暴露物的数量众多,含量和理化性质差异显著,据估计,人体暴露在超过40万种的化学物质中,其中约有5000种外源性化学物质在体内分散和积累^[8,9]^,且它们在体内的含量差异很大(含量差异可达5~6个数量级)。一般而言,环境污染物的含量为10^-15^~10^-6^mol/L,而内源性代谢物的含量为10^-9^~10^-3^mol/L^[10]^,不同物质的巨大含量差异对分析仪器的灵敏度和动态范围提出了巨大挑战。

色谱-质谱联用技术兼具色谱的高效分离能力和质谱的高分辨、高灵敏度特性,已广泛用于暴露组学研究。暴露组学研究方法主要包括靶向定量分析、可疑物筛查和非靶向筛查,图1显示了这3种方法对外源性化学物质的研究层次。靶向定量分析的研究对象被称为“完全已知物”,即化学名称和结构是已知的且在样本中存在的外源性化学物质;可疑物筛查的研究对象被称为“已知的未知物”,即化学名称和结构已知、但不确定样本中是否存在的外源性化学物质;非靶向筛查的研究对象被称为“未知的未知物”,该方法用于发现新的外源性化学物质^[11]^。靶向定量分析通常可以采用三重四极杆质谱实现,可疑物筛查和非靶向筛查则主要采用高分辨质谱实现^[11]^。

**图1:**
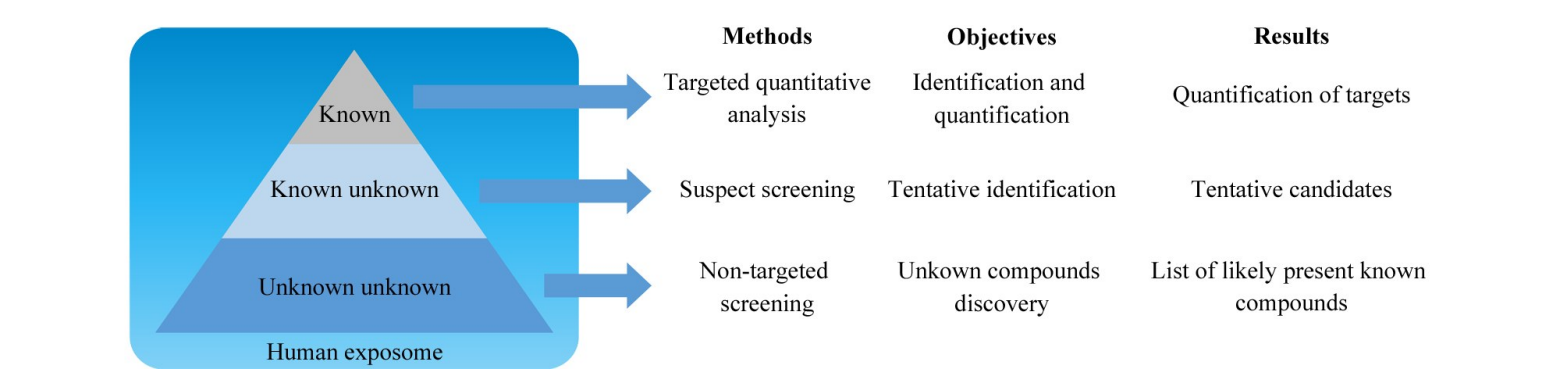
暴露组学分析方法组成

### 1.1 基于色谱-三重四极杆质谱的靶向定量分析

在人体内暴露研究中,各种环境污染物在人体中的残留情况是人们首要关注的。基于三重四极杆质谱和多反应监测(MRM)模式的靶向定量分析是测量内暴露最常用的方法。靶向定量分析需要先确定目标分析物,再使用高选择性的样品制备方法以最大限度地去除基质干扰^[12]^,之后利用高特异性和高灵敏度的三重四极杆质谱在MRM模式下对样品中的目标物进行准确定量及风险评估。靶向定量分析具有灵敏度高、准确度高、通量高的优点,主要体现在以下几个方面:(1)基于三重四极杆质谱结合MRM的靶向分析方法可以在最佳质谱条件下对每一个目标物进行分析,从而显著提高检测灵敏度;(2)该方法通过母离子和子离子的两级离子选择,排除了大量干扰离子,使质谱的化学背景降低,能够显著提高目标检测物的信噪比,从而提高检测的灵敏度;(3)该方法基于标准品建立,实际样本的保留时间、母离子、子离子等多个分析特征已与标准品进行了匹配,检测结果具有高准确度;(4)随着质谱扫描速度的不断加快和动态MRM技术的使用,靶向定量分析技术的通量也在不断提高。

基于气相色谱-串联质谱(GC-MS/MS)和液相色谱-串联质谱(LC-MS/MS)的暴露组学分析方法常被用于监测人体血液(全血、血清或血浆)和尿液样本中的有机污染物。GC-MS/MS方法可用于检测多环芳烃、多氯联苯、多溴联苯醚、多溴联苯、二恶英、多氯二苯并呋喃和一些农药(有机氯农药、有机磷农药、氨基甲酸酯农药和拟除虫菊酯农药)^[13⇓-15]^;其中农药和多环芳烃在血液和尿液中均有检出,但因其蓄积性较弱优先选择尿液作为生物样本;而其他类别的污染物因蓄积性更强优先选择血液作为生物样本。LC-MS/MS方法可用于检测杀菌剂、烟草暴露标志物、邻苯二甲酸酯(PAEs)、环境酚、全氟化合物(PFASs)、有机磷酸酯(OPEs)、紫外线吸收剂、对羟基苯甲酸酯以及挥发性有机污染物(VOCs)和它们的代谢物等^[16⇓-18]^;其中,PAEs、环境酚、对羟基苯甲酸酯和VOCs在体内代谢较快,通常以代谢物的形式在尿液中被检出。PFASs作为近年来受到广泛关注的持久性有机污染物,其在人体内的蓄积性强,半衰期可达数十年之久^[19]^,因此常采用血液样本中的PFASs含量来表征人体对这类化合物的暴露情况。

目前,生物监测方法集中于测量单一类别的外源性化学物质,例如邻苯二甲酸酯代谢产物^[20]^、环境酚类化合物^[21]^、PFASs^[17]^以及有机磷酸酯^[16]^等。同一类别的外源性化学物质具有相似的物理化学性质,与多类别外源性化学物质同时检测的方法相比,同类别外源性化学物质的检测方法更容易确定最佳提取和定量条件。然而,人类每时每刻都暴露在成千上万种化学物质之中,如果对这些化学物质按照类别进行逐一分析,将会花费大量的时间和金钱,并且还可能会受限于样本量而无法对人体暴露进行全面监测,这一缺陷在基于大规模流行病学的EWAS研究中尤为明显。为了解决这一问题,You等^[22]^和Wang等^[23]^将知识导向和基于实际样品的可疑物筛查技术相结合,锁定了与人体暴露相关的外源性化学物质,并建立了包含多类别化学残留物的暴露组学精准定量方法;该方法采用基于96孔除磷脂板的前处理技术,在去除基质效应的同时提高了分析通量。另外,有研究^[24]^更加追求方法覆盖度,在一个靶向方法中同时监测1000种以上的外源性化学残留物,其主要关注的外源性化学物质类别是生物毒素、杀虫剂和兽药等。上述方法均为研究暴露与疾病之间的关联提供了有力支持。

### 1.2 基于色谱-高分辨质谱的可疑物筛查分析

基于色谱-高分辨质谱的暴露物筛查方法主要包括可疑物筛查和非靶向筛查。可疑物筛查是一种对可能存在的已知化合物进行筛查的方法,该方法的目的是大规模快速鉴定复杂混合物中的化合物成分,为进一步的靶向定量分析提供优先监测的化学物质目录^[25]^。可疑物筛查方法需要依赖参考数据库进行定性分析,从而实现对可疑物的筛查^[11]^。以真菌毒素母体及其修饰产物的筛查为例,可疑物筛查方法的主要工作流程如图2所示^[26]^。该方法主要通过比较样品与标准品参考数据库中已知真菌毒素母体及其修饰产物的前体离子质量、保留时间、同位素分布和碎裂方式,从包含众多特征的高分辨质谱数据中筛选出可疑信号^[26]^。Wang等^[27]^通过自建数据库中的母离子、二级特征碎片离子和保留时间信息,实现了对血清中1210种农、兽药(包括部分人畜共用药物)以及其他化学污染物和代谢物的高覆盖筛查,所建立的方法稳定、可靠,适用于大规模血液样本的暴露组筛查,能够在24个混合血清样本的示例研究中筛查出58种外源性残留物。相比于其他非靶向筛查方法,可疑物筛查被认为是多类组分分析方法的延伸,其在分析过程中可以准确地鉴定出部分代谢物,并根据靶向方法进行定量分析。

**图2:**
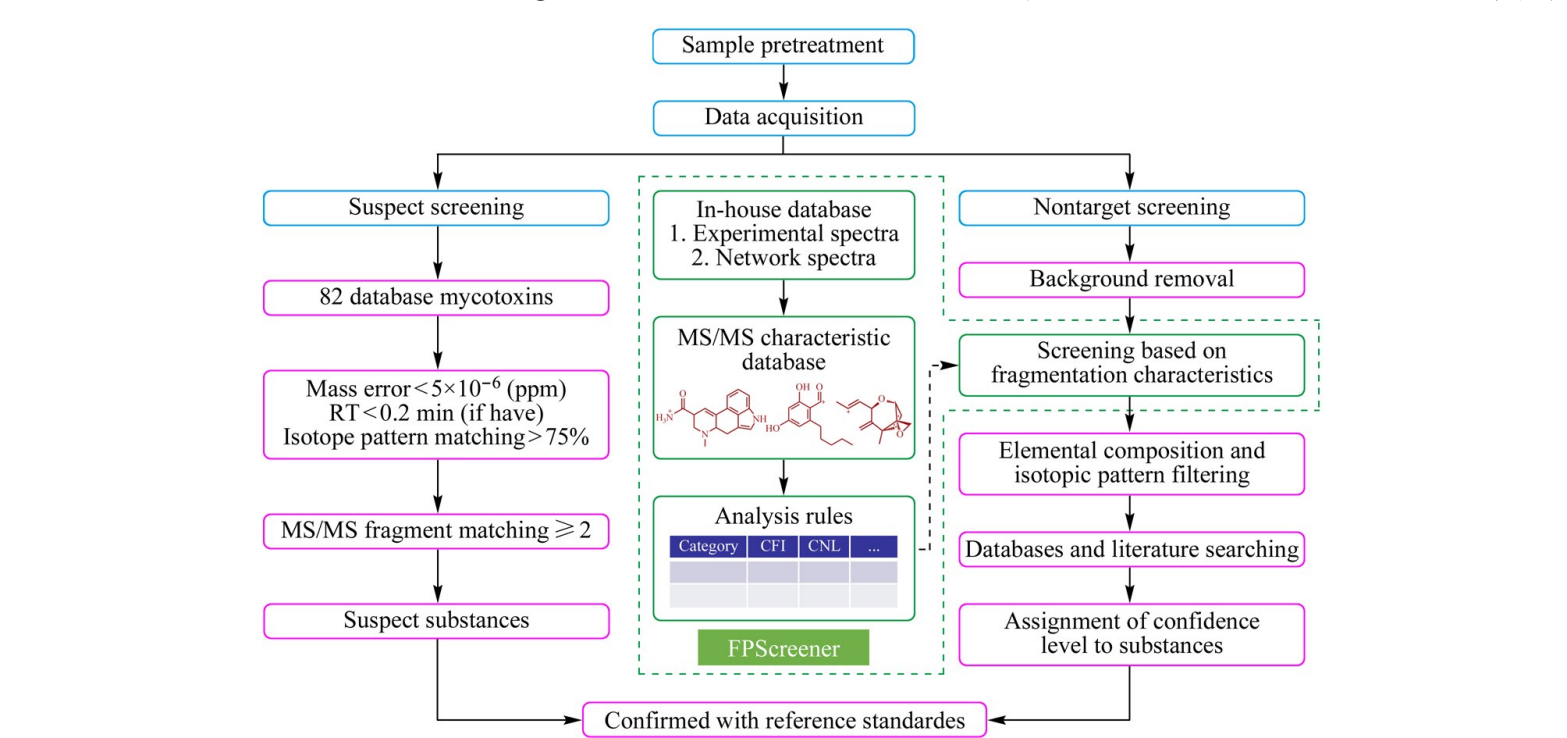
基于高分辨质谱的可疑物筛查及非靶向筛查方法的工作流程^[26]^

### 1.3 基于色谱-高分辨质谱的非靶向筛查分析

在没有明确研究对象的情况下,基于高分辨质谱的非靶向筛查是人体内暴露筛查和测量的重要手段。非靶向筛查是对未知化合物进行筛查的方法,其目的是发现完全未知的化合物,以进一步了解人类所暴露的化学物质。基于色谱-高分辨质谱的暴露物注释结果可以分为5个等级^[28]^,置信水平从高到低依次为(1)Level 1(确切的结构):将实验数据与化合物标准品的保留时间、一级与二级谱图进行匹配,最终得到确切的结构;(2)Level 2(可能的结构):包括Level 2a和Level 2b两类,前者利用实验所得谱图与文献或数据库中的谱图进行比较,从而得到可能的结构,而后者则通过将实验数据与二级谱图中的诊断离子、电离规律、前体化合物信息相结合,得到没有标准品或文献信息的可能的结构;(3)Level 3(初步候选物质):利用实验所得到的一级与二级谱图,推断出化合物可能存在的结构;(4)Level 4(确切的分子式):将化合物精确质量与同位素及离子加合规律相结合,确定化合物的分子式;(5)Level 5(精确质量):利用高分辨质谱所得到的质荷比数据,直接获得目标化合物的精确质量。如图2所示,与可疑物筛查方法相似,非靶向筛查同样采用了简单的样本预处理步骤,并利用高分辨质谱全扫描模式来获得包含成千上万个特征离子的高分辨质谱数据;但二者在数据挖掘和分析方面有很大不同,非靶向筛查没有预先设定的化合物标准品和列表,而是通过研究现有谱图总结出化合物的碎裂特征,并根据碎裂特征、元素组成以及同位素分布模式对去除背景的样品信号进行筛查,之后再通过数据库及文献检索的方式进行组分鉴定,从而筛查出未知化合物。我们课题组^[26,29⇓⇓-32]^针对食品基质中未知或不可预知的风险物质,建立了一系列非靶向筛查技术;其中,针对有空白对照的样品,Fu等^[29]^建立了基于自建数据库的非靶向筛查方法,同时结合特定物质的特征结构碎片,详细解析了二级质谱特征,实现了对自建数据库中没有覆盖到的风险物质的识别;而针对无空白对照的样品,Fu等^[30]^又提出了一种基于平均值偏差倍率计算及特征片段结构检索的潜在风险物质快速锁定方法,并通过自编程序实现了自动化的数据检索和风险化合物锁定,该方法无需分组,可快速筛查、准确测定食品中的潜在风险物质。考虑到风险物质在体内的代谢过程,Liang等^[31]^开发了一种针对复杂食品基质中已知和未知兽药及其代谢物的非靶向筛查方法,构建了包含3710种兽药及其相应代谢物的质谱数据库,归纳了共有或独有的质谱碎裂特征,并基于质谱碎裂特征及所开发的智能检索程序,将其示范性地应用在蛋类样本的风险物质筛查中。南京大学韦斯团队^[33⇓-35]^提出了新污染物的非靶向智能分析新方法,构建了基于多模态分子关系网络的污染物筛查及基于深度学习的谱图-分子结构生成等新污染物分析技术,实现了真实环境中新污染物的高通量精准识别。

综上所述,靶向定量分析、可疑物筛查和非靶向筛查是暴露组学分析中最主要的方法。靶向定量分析采用基于三重四极杆质谱的MRM数据采集模式,可疑物筛查和非靶向筛查采用基于高分辨质谱的数据依赖采集模式,且靶向定量分析在检测灵敏度及定量准确度方面优于另外两种方法;靶向定量分析可实现目标化合物的精准定量,且其数据处理过程更加简单,而可疑物筛查与非靶向筛查均是基于高分辨质谱的半定量数据。然而,相比于可疑物筛查和非靶向筛查方法,靶向定量分析的化学物质覆盖度有限,而基于高分辨质谱及数据依赖采集模式获得的数据可包含成千上万个化学物质特征。在定性分析方面,靶向定量分析依赖于标准品,只能针对已知目标化合物进行分析,进一步限制了检测覆盖度;可疑物筛查依托于标准品数据库,在保证定性准确度的同时,进一步扩大了检测覆盖度;非靶向筛查则不依赖于标准品,它的数据处理方式相对复杂,不同筛查规则对定性结果的准确度影响很大,但非靶向筛查能够发现新的化合物,并提供更全面的化学暴露知识。总之,上述3种方法各有优缺点,实际应用中应根据不同需求选择合适的方法。

## 2 暴露组学研究范式

除了对个体所暴露的外源性化学物质进行全面检测外,对这些化学物质与疾病风险的关系进行评估是暴露组学研究的另一重要方向。因此,本文综述了目前暴露组学的研究范式,包括暴露组关联研究、混合暴露研究、暴露组学与多组学关联研究。

### 2.1 暴露组关联研究范式

EWAS是参照GWAS所提出的,是一种数据驱动的探索性研究范式,可用于发现与复杂疾病相关联的环境因素。目前,暴露组关联研究已成为环境与健康科学领域的重要研究方向^[36]^。通常将暴露组关联研究与流行病学方法相结合,以人群队列为基础,先根据已有研究确定暴露变量和疾病结局,再利用统计方法筛选出与疾病有显著关系的暴露变量,从而在暴露变量中识别出重要的暴露因子。

目前暴露组关联研究所涉及的大型队列主要有美国营养与健康调查(NHANES)、加拿大健康测量调查(CHMS)、欧洲人类早期暴露计划(HELIX)、韩国国民健康与营养调查(KoNEHS)队列等^[37]^。研究过程中涉及的内部暴露变量包括微量营养素(如维生素)、代谢物和蛋白质(如脂肪酸和C-反应蛋白等)、污染物(如重金属、酚类化合物、持久性有机污染物、氟化物、有机磷农药和内分泌干扰物(EDCs)等),这些暴露变量都是通过血液和尿液中的生物标志物来测定的。暴露组关联研究在关注上述内部暴露变量对健康影响的同时,还揭示了生活方式、大气环境、社会因素等外暴露因素与疾病存在的密切关系。研究过程中涉及的疾病,包括哮喘^[38]^、癌症^[39]^、不良妊娠^[40]^和发育异常^[41]^等,都被证明与特定环境暴露密切相关。除此之外,一些慢性疾病也在暴露组学关联研究中被关注,例如糖尿病、高血压、高尿酸、高血脂、肥胖等^[22]^。在对实验数据进行统计分析时,常采用广义线性回归模型,并根据结果变量进行回归方法的选择。当结果变量为二分类变量时,采用逻辑回归;当结果变量为连续变量时,则采用线性回归或加权线性回归。此外,在统计分析过程中,常将协变量纳入模型以对模型进行调整,同时为了控制结果的假阳性率,一般采用多重检验进行模型校正。

暴露组关联研究可以评估多种环境因素与疾病之间的关系,为揭示环境因素对健康的影响提供了重要的科学依据。然而,该方法仍存在一些局限,其仅能表征暴露因素与不良结局之间的相关关系,而非因果关系,需要结合前瞻性队列、毒理实验以及基因组学、分子生物学等联合分析来对因果关系进行进一步确认。

### 2.2 混合暴露研究模型

暴露组关联研究侧重单一化学物质或一组结构类似化学物质的健康影响分析,而很少关注化学混合物的“鸡尾酒效应(cocktail effects)”。但实际上,人体处于多种污染物的联合暴露之中,为了解决这一难题,近年来已经出现一些混合暴露研究模型,用于评估多个环境因素对健康的共同影响,并筛选出对健康结局具有显著影响的因素;其中最具代表性的模型是有加权分位数和回归模型(weighted quantile sum, WQS)^[42]^、分位数-G-计算模型(quantile g-computation, Q-gcomp)^[43]^、贝叶斯核机器回归模型(Bayesian kernel machine regression, BKMR)^[44,45]^、最小绝对收缩和选择模型(least absolute shrinkage and selection operator, LASSO)^[46]^以及删除/替换/添加模型(deletion/substitution/addition, DSA)^[47]^。

WQS模型于2015年由Carrico等^[42]^开发,该模型的基本原理是构建一个加权指数,用以估计所有预测变量对健康结果的混合效应,同时通过在回归模型中构建相关协变量来检验该指数与因变量或结果的关联。相比于暴露组关联研究,WQS模型不仅可以评估暴露混合物对健康的影响程度,还能在高度相关的外源性化学物质中识别出对健康影响更大的变量^[42]^。Caporale等^[48]^利用WQS模型建立了混合暴露与儿童语言延迟之间的关联,选出了与健康具有显著关联的内分泌干扰物,并对儿童性别、母亲吸烟状况、胎次、鱼类消耗、母亲受教育程度和肌酐浓度等潜在混杂因子进行了调控。

Q-gcomp模型是一种用于估计混合物联合效应的新方法,于2020年由Keil等^[43]^在WQS模型的基础上开发。该模型结合了g计算(一种因果效应估计方法),能够进一步提高模型性能。Q-gcomp模型的基本原理是评估当所有暴露变量的含量同时增加一个分位数时疾病风险增加的比例。与WQS模型相比,Q-gcomp的计算速度更快,无需像WQS模型一样对于正相关和负相关效应进行分别计算,而且可以在一个模型中同时评估所有混合物的效应。此外,对于小样本数据,Q-gcomp模型能够展现出更强的鲁棒性^[43]^。

BKMR模型于2015年由Bobb等^[44]^开发,可用于估计混合物的健康效应,为了使该方法易于使用,该研究团队又在2018年开发了基于R编程语言的开源软件包^[45]^。BKMR模型将暴露变量作为自变量、健康结局作为因变量,通过建立平滑函数h来评估暴露因素对健康的影响,同时BKMR模型还支持混淆因素的调整。BKMR模型支持变量选择功能,能够确定组分对混合物健康效应的贡献大小;同时,该模型还支持层次变量选择功能,即结合先验知识对混合物进行分组,解决了混合物组分的共线性问题。因此,利用该模型,能够获得混合物的总体效应、每个污染物的单独效应、每个或每组污染物的重要程度(PIPs)、每个污染物和健康结局的剂量-反应曲线以及污染物之间的交互作用。

LASSO模型是一种用于筛选变量和降低模型复杂度的方法,该模型可用于确定对健康结局影响较大的一系列化学物质。LASSO模型本质上是一种广义线性回归模型,它的基本原理是在传统线性回归模型的损失函数中引入惩罚项(L1正则项),通过压缩回归模型中的变量系数来进行变量选择。相比于将所有变量都纳入模型的回归分析,LASSO模型可以有选择性地去除对结局影响较小的变量,从而降低模型的复杂程度,避免模型的过拟合现象。LASSO模型在暴露组学研究中已有应用,Soomro等^[49]^在一项探究外源性化学物质暴露与妊娠高血压关系的前瞻性队列中,利用LASSO回归模型进行关键暴露变量的筛选,发现邻苯二甲酸单乙基酯和全氟壬酸是与妊娠高血压现象最相关的化学物质。

DSA模型也是一种变量选择模型,它通过迭代的方式来实现多种暴露变量的筛选,主要包括以下3个步骤^[50]^: (1)构建模型空间,即在给定条件下,构建由基础模型线性组合而成的整个模型空间,利用最高阶相互作用以及最大“幂和”来确定候选预测变量的基础模型,同时指定出模型尺寸的最大值;(2)搜索模型空间,即从截距模型开始迭代搜索模型空间,并在每一轮迭代过程中进行预测变量的删除、替换以及添加操作,直至模型尺寸超过设定的最大值;(3)基于交叉验证选择模型,即通过交叉验证筛选出预测方程均方根误差最小的模型及其所包含的预测变量,从而实现暴露变量的筛选。DSA方法于2004年由Sinisi和van der Laan提出,最初被应用于基因组学研究中转录因子结合位点的寻找^[51]^,之后也被用于涉及多种外源性化学物质的环境研究中^[50]^。例如,Nieuwenhuijsen等^[52]^利用DSA模型在60个环境暴露因素中发现了公交路线、景观多样性和交通密度与婴儿出生体重之间有显著关联。

上述混合暴露模型中,WQS、Q-gcomp、BKMR主要用于评估混合物对健康结局的综合影响,LASSO和DSA模型侧重寻找多个环境因素中对健康结局影响更大的环境因素。目前已有将多种模型结合用于联合分析的案例,未来这些模型的不断发展和改进将有利于更好地理解复杂环境混合物对健康的影响,并获得更准确和全面的科学依据。

### 2.3 暴露组学与多组学关联研究范式

目前,研究者们已经开发出了将暴露组学研究与基因组学、转录组学、蛋白质组学、代谢组学等组学手段相结合的全新研究范式,其中基因组与暴露组的结合有助于揭示暴露因素对疾病风险的因果关系。实现这种因果推断的一个重要方法是孟德尔随机化(Mendelian Randomization, MR),该方法将与暴露因素具有强相关的遗传变异作为工具变量,以评估暴露因素与结局之间的因果关系^[53]^。应用MR方法必须满足以下三大假设^[54]^: (1)基因组变量的单核苷酸多态性(SNP)与所研究的暴露因素之间具有强相关性;(2)SNP与混杂因素无关;(3)SNP只能通过暴露因素对结局产生作用。由于基因组与健康结局有明确的因果关系,因而在暴露组关联研究中引入基因组可以有效地解决反向因果问题。MR方法已在近期研究中得到应用,如Choi等^[55]^利用MR方法评估了106个环境因素与抑郁症之间的潜在因果关系,结果发现,社交、睡眠、媒体、饮食和运动相关领域的多种暴露因素与抑郁症存在前瞻性关联。Huang等^[56]^利用MR方法评估了砷暴露与慢性瘙痒症之间的因果关系。

转录组与暴露组的结合有助于揭示环境因素对基因表达水平的影响,从而更好地理解暴露对特定基因表达影响的机制。随着RNA测序技术的发展,通过一次测序得到千万条以上序列的高通量分析已被实现,根据定量基因表达数据又可以进一步实现差异表达基因的发现、富集分析和功能预测。转录组与暴露组的结合在探究环境暴露效应方面已有应用案例,Li等^[57]^以人类胚胎干细胞诱导分化的视网膜类器官为模型,利用暴露组和转录组技术揭示了低剂量多溴二苯醚暴露对人类早期视网膜发育的影响,其中通过转录组分析发现了类器官在经过多溴二苯醚暴露后产生的一系列差异表达基因,从而确定蛋白质消化吸收和细胞外基质受体相互作用是受暴露因素影响的重要途径。

蛋白质组与暴露组的结合有助于明确与暴露因素相关的蛋白质分子特征,从而揭示它们之间的潜在相互作用。蛋白质组学研究包括蛋白质表达水平、翻译后修饰、蛋白质结构与功能、蛋白质之间的相互作用等。质谱技术是目前蛋白质组学分析最常用的技术,其可以高通量地定量蛋白质组。Luminex技术是基于高通量微孔板的多重检测抗体芯片技术,也被用于蛋白质的分析。Gao等^[58]^基于一个纵向人群队列,利用液相色谱-高分辨质谱联用技术和Luminex技术分别开展了非靶向蛋白质组学分析,同时利用液相色谱-串联质谱技术开展了暴露组学分析。随后,研究人员通过关联研究发现了与外源性化学残留物显著相关的蛋白质和相关信号通路;其中免疫相关途径是与暴露组最高度相关的信号通路之一,说明免疫系统在对外来化学物质的应答和调节中起到了重要作用。Maitre等^[59]^基于人类早期生命暴露组项目中由1301对母子组成的多中心队列展开多组学特征研究,并利用Luminex技术测定了血浆中36种细胞因子、载脂蛋白和脂肪因子,并通过探究这些蛋白质与外源性化学残留物之间的关联,发现了肥胖儿童血液中亲脂性持久有机污染物与由脂肪组织产生的蛋白质密切相关。

代谢组与暴露组的结合有助于揭示由环境因素引起的体内代谢扰动机制。借助于质谱技术的进步,代谢组学分析的通量不断提高,在环境暴露与不良健康效应关系研究中的应用潜力也迅速增加^[60]^。将代谢组学方法应用于暴露风险分析,可以揭示生物体在受到环境因素影响后,其体内代谢产物的组成、含量以及所处代谢通路的变化等信息。近年来,代谢组学方法已在基于人群队列的污染物健康效应研究中得到了广泛应用。Liang等^[61]^为了评估交通相关空气污染暴露对人体分子通路造成的不良影响,对45名正常通勤者和患有哮喘通勤者的血液样本进行了高分辨代谢组学分析,测定了27种空气污染物的含量,并对这些污染物相关的代谢物进行了代谢通路分析;结果发现,在患有哮喘的通勤者体内,几种炎症相关的代谢通路和氧化应激相关的代谢通路均发生了改变,其中精氨酸、组氨酸和甲硫氨酸是与空气污染相关的关键代谢物,这一发现更好地揭示了交通相关空气污染物对哮喘病人的潜在不良影响。Alderete等^[62]^结合代谢组学方法和通路富集分析,揭示了与血浆中PFASs浓度相关的代谢紊乱;结果发现,较高水平的PFASs暴露与几种脂质和氨基酸通路的代谢紊乱以及西班牙裔青少年血糖稳态的长期变化之间存在紧密关系。在一个母婴队列中,Wu等^[63]^利用代谢组学分析结合中间相遇方法,发现了多种代谢物可以作为金属或类金属元素与妊娠糖尿病之间关联的标志物,这些代谢标志物主要涉及脂质代谢和腺苷酸/精氨酸/一氧化氮代谢途径。You等^[22]^利用代谢组学和中间相遇方法分析了PFASs暴露与高尿酸血症风险正相关关系背后的代谢扰动,发现脂质代谢物是介导该过程的重要代谢物。此外,Wang等^[23]^通过分析电子垃圾拆解地区及临近非暴露地区的孕妇胎盘组织发现,处于电子垃圾拆解地区的孕妇暴露了大量的多溴联苯醚,而这些多溴联苯醚与新生儿头围和1 min内肤色、心率、对刺激的反应、肌张力和呼吸综合评分(appearance, pulse, grimace, activity, respiration score at 1 min, Apgar1)值的降低显著相关;其中参与该过程的代谢途径有磷酸戊糖途径、抗坏血酸代谢途径、苏氨酸代谢途径、丁酸代谢途径、脂质代谢途径和精氨酸生物合成等。总之,将代谢组与暴露组相结合,能够系统性地揭示环境暴露后的机体代谢紊乱现象,为研究环境暴露引起的疾病机制提供额外的见解。

将暴露组学与基因组学、转录组学、蛋白质组学、代谢组学等组学分析方法相结合,有助于在多个生物学层面揭示环境因素对生物体的影响机制。多组学整合分析能够综合利用高维分子测量与计算技术,阐明生物体内的复杂相互作用,帮助揭示环境因素引起的生物学变化,并进一步评估环境因素对健康的影响^[64]^。目前,已有不少研究利用多组学整合策略来研究环境因素对健康的影响。Chao等^[65]^通过测量胎盘组织中多种内、外源性化学物质,结合表观基因组和转录组,发现一些外源性化学物质与子痫前期相关的分子特征有很强的相关性,这一结果表明外源性化学物质可能影响表观基因和转录过程,揭示了子痫前期的潜在发病机制。另一项研究^[66]^基于HELIX队列探究了早期生活中的环境暴露对生命周期健康影响的分子表型;在研究过程中,研究人员将在妊娠期和儿童期暴露组学研究中发现的一百多种暴露因素(化学物质、户外、社会和生活方式)与儿童期的多组学特征(甲基化组、转录组、蛋白质组和代谢组)相关联,发现了多种暴露因素和分子特征之间存在显著关联,揭示了早期生活环境暴露中潜在的生物反应和暴露源。总之,通过整合分析多个不同层次的生物学信息,有助于深入了解不同分子层面的环境暴露与健康之间的关联,并为健康风险评估提供更准确和更全面的科学依据。

上述方法各有优缺点,暴露组关联研究的优点是模型简单,容易确定外源性化学物质和结局的关系,是目前最常用的暴露组学研究方法,但目前相关研究大多是基于横断面人群队列开展的,无法获得暴露因素和结局之间的因果关系。此外,暴露组关联研究不考虑化合物之间的相互作用,容易导致虚假关联的产生。混合暴露研究的优点是可获得多种外源性化学物质的联合暴露效果,相比于单变量暴露研究,混合暴露研究更加接近真实世界的暴露场景。然而,目前的混合暴露模型有限,在纳入变量较多的情况下很容易造成模型过拟合。暴露组与多组学关联研究的优点是可以发现外源性化学物质与多种生物分子特征之间的关系,能够更加深入地探究暴露因素对生物体的作用机制,但其技术门槛和实验成本较高,开展难度大。目前混合暴露研究和暴露组与多组学关联研究仍未得到广泛应用,但可以预见的是,随着数据技术和分析方法的不断进步,未来这些研究范式将在环境健康研究中发挥越来越重要的作用。

## 3 总结与展望

近年来,组学技术的进步为暴露组研究带来了空前的发展,暴露组的内涵和外延也得到了完善和更新。然而,在现有条件下仍然无法准确定量一个人的暴露组,因此暴露组研究所采用的分析技术和方法仍需进一步完善。结合实验室近期工作,对暴露组学的未来做出如下展望:(1)在方法学层面,暴露组学关注的是所有环境因素的总和,然而现有的研究着重于人体内暴露,难以对暴露组进行全面表征。因而未来需要更高覆盖度的方法来全面监测人体内、外暴露,并将二者结合用以全面阐明环境因素对人类健康的影响。(2)在暴露因素与不良健康效应的关联研究中,尽管利用大规模人群数据获得了较稳健的关联结果,但仍然可能存在一些未被测量的混杂因素,导致关联结果的准确度受到影响。所以未来研究中应考虑更多的混杂因素,在更大规模的人群研究中控制混杂因素,进一步提高关联结果的稳健性。(3)目前,横断面研究方法在暴露组学研究中被广泛使用,然而这一方法不能提供暴露因素与不良健康效应间的因果关系。因此,未来应该开展大规模的前瞻性队列研究,对已发现的暴露疾病风险关联结果进行因果关系验证。(4)关于暴露因素对慢性疾病风险的作用机制,需在基因组、蛋白质组和代谢组等多层面上进行探究,才能窥见环境暴露对慢性疾病影响过程的全貌。结合系统生物学和环境毒理学等多学科,共同深入阐明暴露因素对慢性疾病发展风险影响的具体作用机制是未来的发展趋势。

## 作者团队简介

中国科学院大连化学物理研究所生物分子高分辨分离分析及代谢组学研究组(1808组)隶属于国家色谱研究分析中心、中国科学院分离分析化学重点实验室和中国科学院大连化学物理研究所生物技术部。在许国旺研究员的领导下,多年来一直致力于色谱理论及应用基础研究。根据分析化学的特点和国际前沿研究领域的发展趋势,立足于中国现状,结合国家重大应用领域的需求与自身技术优势,以分离分析研究为立足点,以生命科学、重大疾病、中医药现代化、公共安全等领域的复杂样品分析为切入点,开展极端复杂体系的分析方法学及其应用研究、代谢组学方法及其应用研究和转化医学等工作。

课题组网站: http://www.402.dicp.ac.cn/。

**Figure:**
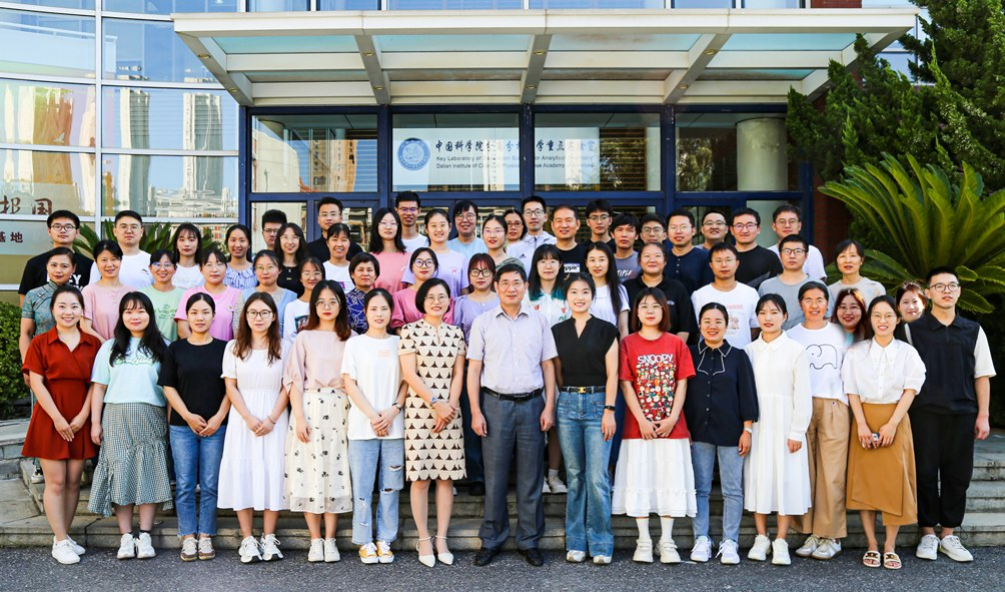


### 人才队伍

**学术带头人 许国旺研究员:** 国家自然科学基金委杰出青年基金项目获得者,中国科学院大连化学物理研究所代谢组学研究中心主任,中国化学会色谱专业委员会主任等

**研究组负责人 刘心昱副研究员: **中国抗癌协会肿瘤代谢委员会青年委员,辽宁省免疫学会生物质谱分会副主任委员

**研究组职工及学生: **固定职工16人,博士后5人,硕、博士研究生30余人

**团队精神: **做“国家事”,担“国家责”

### 科研项目及成果

**科研项目:** 国家科技重大专项,国家重大科学研究计划,国家自然科学基金等

**科研成果:** 构建了一套高灵敏、高覆盖代谢组定性和定量的综合分析技术体系,广泛应用于重大疾病的研究;开创了拟靶向代谢组学技术,首次实现了在单个方法中半定量超两千种代谢物;研发了国际先进的多维色谱-质谱联用技术,单个方法能够定量超千种代谢物,突破了灵敏度和覆盖度的限制;构建了非靶向和靶向暴露组学新方法,分别实现了超千种和超百种污染物的筛查和精准定量;已在*PNAS*, *Nat Protoc*, *Nat Commun*, *Cell Metab*, *Nat Methods*, *Anal Chem*等期刊发表SCI论文540多篇;申请发明专利百余件(授权90多项)

**获奖情况:** 国家科技进步二等奖,辽宁省科技发明二等奖,中国分析测试协会科学技术成果特等奖等

### 研究领域

1. 基于色谱-质谱的极端复杂样品的分析方法及其应用;

2. 代谢组学分析平台及其在疾病生物标志物发现、合成生物学、植物功能基因组、代谢网络分析和重建中的应用,同时关注人工智能的应用;

3. 新暴露方法的发展及其在环境和不良健康影响中的应用;

4. 精准医学中的单细胞和空间代谢组学,专注于癌症、糖尿病、肝病等重大疾病。

### 仪器设备

**实验室:** 质谱实验室,色谱实验室,分子生物实验室等

**仪器设备:** Orbitrap Q-Exactive高分辨质谱, Q-Exactive HF高分辨质谱, LTQ Orbitrap XL高分辨质谱, Zeno 7600高分辨质谱, 5600+高分辨质谱, Qtrap 6500+三重四极杆质谱, Q-TOF 6546高分辨质谱, CE-TOF高分辨质谱, Waters TQ-XS三重四极杆质谱等

**Figure:**